# Preparation of MWCNT Microbeads for the Application of Bed Materials in a Fluidized Bed Heat Exchanger

**DOI:** 10.3390/ma13061289

**Published:** 2020-03-12

**Authors:** Min Ji Lee, Sung Won Kim

**Affiliations:** School of Chemical and Material Engineering, Korea National University of Transportation, Chungju-si, Chungbuk 27469, Korea; mj1226@ut.ac.kr

**Keywords:** carbon nanotube, MWCNT microbead, fluidized bed, *m*-cresol, thermal conductivity

## Abstract

Fluidized beds have been utilized for various chemical and physical applications including heat transfer such as the gas–solid heat exchanger. It is advantageous to use carbon nanotubes (CNTs) with high thermal conductivity as bed materials for heat transfer enhancement in a direct gas–solid contacting heat exchanger. However, the poor fluidization of CNTs is the biggest challenge due to the strong cohesive force between the particles. A control over the macroscopic shapes of CNT powders is required for their application. A preparation method of CNT microbeads has been proposed to be suitable for fluidized bed applications. The method is characterized by using *m*-cresol known as processing solvents for fabrication of the CNT microbeads. Multiwalled CNT powders were directly mixed with *m*-cresol to yield a thick paste-like material. The paste droplets were rolled into round particles with in pure water with and without surfactant. The obtained particles were dried in a vacuum oven. The obtained microbeads have diameters ranging 300–2200 μm and apparent particle density of 350–400 kg/m^3^, which corresponds to Geldart group B in the fluidization classification. The micrograph of the CNT microbeads exhibited stacked nanotubes array on the surface, indicating obvious densification of the raw CNT powders. The microbeads prepared in water containing surfactant have better shape factor such as circularity and solidity. The thermal conductivity of the microbeads is about 1.18 W/mK in a bulk state, which is much higher than raw CNT powder (0.032 W/mK). The flowability and fluidization characteristics of the multiwalled CNT (MWCNT) microbeads showed a possibility as promising bed material suitable for the fluidized bed heat exchanger.

## 1. Introduction

Fluidization is a physical phenomenon that makes solid particles into a fluid-like state using a fluid medium such as a gas or liquid [1]. The fluidized bed has been in existence as a reactor type using the fluidization technology for more than a century. The fluidized bed reactors have been utilized for fluid catalytic cracking, combustion, heat or mass transfer such as gas–solid heat exchange and solid coating [2,3]. Recently, nanoparticles with high specific area and reactivity have played an important role in various powder-based process areas. Due to the mentioned reasons, there have been growing interests in an effective application of the nanoparticle in the fluidized bed in the last decade [4].

Carbon nanotubes (CNTs) among various nanoparticles have been mentioned as attractive materials for application in the fluidized bed because of their excellent thermal and electrical conductivities and mechanical strength [5]. Since the fluidized beds have high rates of heat and mass transfer, it could be advantageous to use the CNTs with high thermal conductivity as bed materials for heat transfer enhancement in direct gas–solid contacting heat exchanger such as air or nitrogen preheater.

However, the strong anisotropy of the nanotubes on the CNT powder surface can affect bed behavior of the particles in the fluidized bed [6]. It is well known to be difficult to fluidize the CNTs in a fluidized bed since the interparticle attraction force is stronger than fluid mechanical movement during the handling of CNT powders [7,8]. It is believed that this can be overcome by a control over the macroscopic shapes of CNT powders for their practical application in the fluidized bed. In particular, a formation of large size particles with hundreds micrometer scale will be required for the CNT application as a bed material having high thermal conductivity and heat capacity in a fluidized bed heat exchanger [9]. In addition, a preparation of rounded particles should be considered to minimize inter-particular attrition caused by vigorous particles movement in the fluidized bed [10].

A macronization of nanoparticles, such as the CNT particles, can generally be achieved by the solvent-based strategies for dispersion and processing of the particles. However, some types of solvents for the direct dilution such as 1,2-dichrolobenzene [11], dimethylformamide (DMF) [12] and N-methyl-2-pyrrolidone (NMP) [12] can disperse the nanotubes less than 0.02 wt %, indicating extensive solvents treating and uneconomical efforts in the process of the CNT macronization [13]. Most of the studies have used single-walled CNTs (SWCNTs) for their application in the electronic device [11,12,13].

Although various attempts on preparation of the macronized CNT particles have been made up to now, most of the studies have been directed to the highly porous aerogel with low density [14]. Research on the preparation of dense CNT particles ranging in the size from several hundred microns to several millimeters for the fluidized beds is comparatively sparse [14].

Recently, Kang and Moon [15] reported a preparation method for the multiwalled CNT (MWCNT) macronization in the form of ball with hexadecane and surfactant for electric device application. However, the size of the obtained particles with density of 100–300 kg/m^3^ was in the range of 8–12 μm, which belongs to group C in the Geldart classification [16] and are not suitable for fluidized beds due to strong van der Waals force [2]. An alternative method to prepare denser and bigger rounded-particles using a solvent, which can disperse the CNT at a high concentration, is required for the fluidized bed process. However, density control of the MWCNT was rarely reported and the work is still challenging [14]. Recently, Chiou et al. [13] reported a CNT dispersion method using cresols as a processing solvent, where *m*-cresol can disperse the CNT powder at high concentration up to tens of wt % without any dispersing agent. Their study demonstrated a few proofs such as thread and film from thick paste, showing a possibility in densification of the CNT powder [13].

In the present study, a preparation method of the CNT microbeads suitable for the fluidization has been proposed to apply the CNT as bed materials in a gas–solid fluidized bed. The method was characterized by increasing the density and size of the CNT particles using *m*-cresol, and forming the rounded particles to be suitable for fluidized bed operation. The basic fluidization properties as well as the physical and thermal properties of the CNT microbeads have been determined for the verification of the prepared particles.

## 2. Materials and Methods

### 2.1. Material

**Figure 1:**
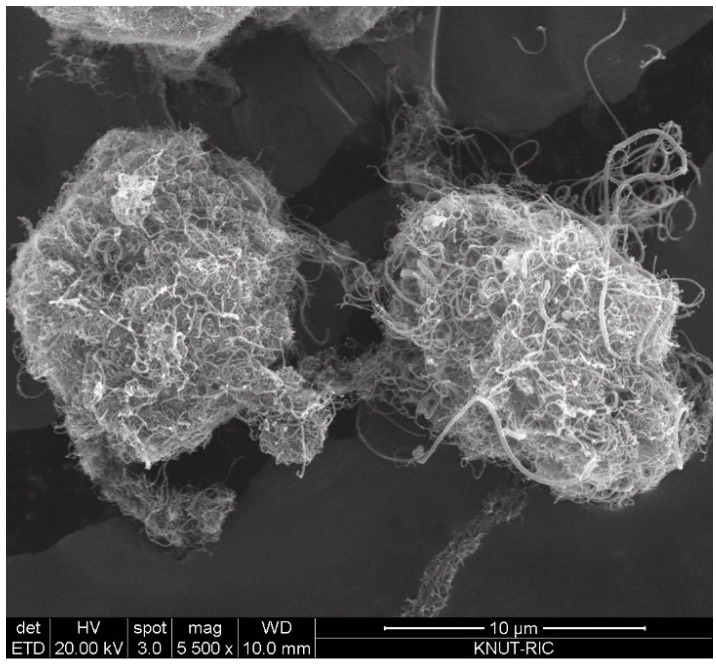
SEM image of vertically aligned carbon nanotube (VACNT) used in experiments.

The CNT used in this study are multiwalled CNT (MWCNT) produced in a fluidized bed reactor, which is based on the method of catalytic chemical vapor deposition (CCVD). The CNT powders (FloTube-7000) were purchased from the Cnano Technology Ltd. (Jiangsu, China). The CNT (average d_p_ = 0.31 mm; bulk density = 19 kg/m^3^) is the vertically aligned CNT (VACNT) as a type of MWCNT and features in long nanotubes grown perpendicular to a particle surface [17]. Particle size of the raw CNT ranges from 0.015 to 1.75 mm depending on the degree of physical entanglement. Figure 1 is SEM (scanning electron microscopy) images to show the typical shape structure of the raw CNT particles. The VACNT is characterized by intertwined particles by long nanotubes and internanotubular entanglement. Since big void space is easily formed inside the entangled VACNTs, the apparent particle density is much small [17]. *m*-cresol (99%) was purchased from Sigma-Aldrich (St. Louis, MO, USA) and used as received.

### 2.2. Method

**Figure 2:**
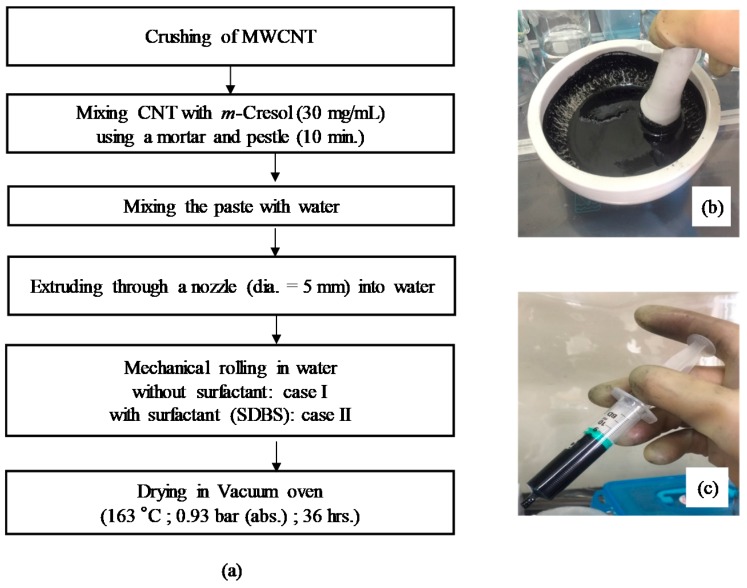
Flow diagram of preparation process of carbon nanotube (CNT) microbeads (**a**); mortar (**b**) and syringe for extrusion (**c**) used in the process.

Figure 2 shows a flow diagram of the preparation process of the CNT microbeads. Crushed powders of the raw MWCNT were directly mixed with *m*-cresol by using a mortar and pestle at a concentration of 30 mg/mL for 10 min. Then, the MWCNT/*m*-cresol paste was mixed with deionized water in the mortar at ambient condition (the mass ratio was paste: water = 1.41:1.00). The initial shape of the CNT microbeads was formed by extruding the CNT paste through a nozzle with an inner diameter of 5 mm into a water vessel, where the water is pure water for case I or water containing 1 wt % of surfactant (SDBS: sodium dodecylbenzene sulfonate) for case II to investigate the effect of surfactant on surface shape of the microbeads [15]. The initial beads were mechanically rolled to make them round in the water vessel. The water vessel containing the initial beads were placed on plate of a shaker (SH30; FINEPCR, Gunpo-si, Korea) and subjected to the mechanical rolling for 10 min at 100 rpm. The initial CNT beads were dried in a vacuum oven at 163 °C and 0.93 bar for 36 h.

### 2.3. Characterization

Images of the SEM were obtained using a Bruker Model Quanta-400 scanning electron microscopy (Billerica, Middlesex, USA). Particle density of the CNT beads was measured by a pycnometer (ASTM D 854-14 [18]). Particle size of the raw CNT was obtained using a particle size analyzer (PSA: LA-950 V2, Horiba, Kyoto, Japan). The average particle size of the CNT beads was measured by a screening method using a sieve screen. Circularity and solidity of the raw CNT and CNT beads were obtained image analysis using the Image J (version 1.50i) [19] after photographing them [6,7,17]. Thermal conductivities of the raw CNT powder and the CNT beads were obtained by using light flash apparatus (LFA 467, Netzsch, Selb, Germany) for thermal diffusivity and ultra-low temperature differential scanning calorimetry (DSC-214 Polyma, Netzsch, Germany) for heat capacity values. Thermo-gravimetric analyses (TGA) of the raw CNT and the CNT microbeads were carried out at a heating rate of 10 °C/min in air by thermo-gravimetric analyzer (TGA-8000, PerkinElmer, Waltham, US). Repose angles of the raw CNT and the microbeads were measured by the method of ASTM C1444 [20]. Fluidization characteristics such as minimum fluidization velocity were determined in a fluidized bed cold model reactor (0.05 m ID) where pressure drop across the bed was measured with varying air velocity [3]. The details of the cold model reactor can be found elsewhere [3].

## 3. Results and Discussion

### 3.1. Physical and Thermal Properties

**Figure 3:**
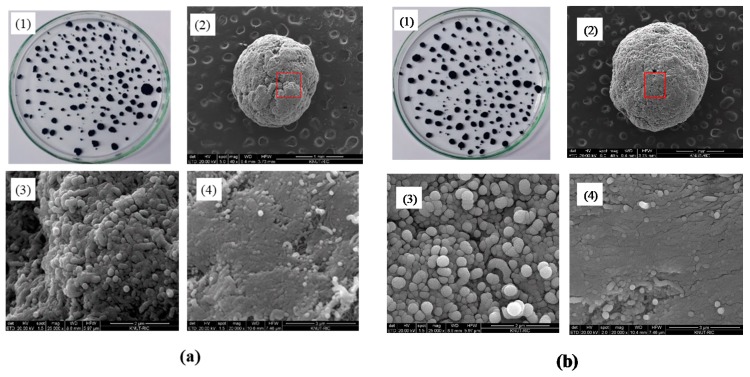
Magnified and SEM images of CNT microbeads: (**a**) Case I and (**b**) Case II; (1) magnified image, (2) SEM image of microbead (scale bar: 1 mm), (3) SEM image of internal pore (scale bar: 2 μm), (4) SEM image of surface (scale bar: 3 μm).

Figure 3 showed macroscopic and SEM images of the prepared CNT microbeads samples of case I and case II. The particles with apparently round shapes and diameters ranging from 0.3–2.2 mm were formed as in the Figure 3(a1,b1). The shapes of both CNT microbeads shown in the SEM images were mostly similar and close to a sphere, but a full sphere bead could not be obtained due to the limitations of the method by mechanical rolling as a preparation step. The surface of the case 2 beads using the surfactant in the preparation looked smoother. Dense regions of nanotubes (Figure 3(a4,b4)) and internal macro-pores (Figure 3(a3,b3)) were observed in the enlarged surface images of the CNT microbeads. In the magnified image of the dense region (Figure 3(a4,b4)), it was identified that compactly stacked regular arrays of nanotubes were formed in the preparation process. This compact array structure would be beneficial for heat transfer applications as bed material in the fluidized bed [21], because a highly dense nanotubes layer can increase thermal conductivity as well as electrical conductivity between the nanotubes [22].

Density is one of the key measures in the macronization of the CNTs, and the density control is affected by various methods such as dispersion and drying in preparation steps [14]. In particular, densification of the MWCNTs has been rarely reported compared to other types of CNTs such as SWCNT (single walled CNT) [14]. Table 1 summarizes bulk density, apparent density and shape information for the raw MWCNT powder and the prepared CNT microbeads. The raw CNTs had a relatively low apparent density due to their very high enveloped-volume, as are the characteristics of the VACNTs with large internal voids [17]. On the other hand, the prepared CNT microbeads showed a result of increasing the particle density by more than 10 times compared to the raw CNTs. In addition, the density value is higher than 100–300 kg/m^3^ obtained from Kang and Moon’s study [15] using the MWCNT. The m-cresol is known to dissolve well highly conjugated polymer such as conducting polymer through second doping [23]. In the same way, the m-cresol interacts with the CNT by charge-transfer through the phenolic hydroxyl proton [13]. By this mechanism, m-cresol initially disperses the nanotubes well at high concentrations to produce a thick CNT paste. Then, when the sized paste droplets are immersed in water, the low water-soluble and hydrophobic paste droplets are coagulated into densification-enhanced beads. The densification of the nanotubes can be seen well in Figure 3(a3,a4) and Figure 3(b3,b4). The density of the case II microbead using surfactant was slightly lower in a comparison between the microbeads, which is thought to be because the surfactant increased the dispersion of nanotubes near the surface and lowered the stacking degree of nanotubes. As already shown in Figure 3(a2,b2), the average particle size was increased about 5–6 times compared to the raw CNT, and the diameter of case II particle using surfactant was larger than that of case 1. It is believed that this increase in particle diameter is dominated by the size of droplets initially formed from the nozzle, but further study will be needed to determine the size of the nozzle and the effect of paste flow through the nozzle.

In analyzing shape information, circularity and solidity are defined as follows [8,19]. The circularity is defined as 4π × area/(perimeter)^2^ of the particle. The closer to a value of 1.0, the closer to a perfect circle, and the lower value indicates an increasingly elongated shape. The solidity indicates the degree of absence of a concave with respect to any shape, representing the roughness of the object. A solidity value close to 1.0 means that only a convex surface is present and the surface is smooth. The circularity was greatly increased by the microbead formation of the CNT powder, and the solidity was also increased. Interestingly, the case II beads showed a higher value than case I. It is believed that the surfactant weakens the coagulation of the nanotubes on the surface of initially formed microbead, thereby providing an opportunity to smoothly recoagulate the surface shape of the bead in the mechanical rolling step (Figure 3(b2)). On the other hand, case I dropped in pure water is difficult to form sphere due to low spherical drop formation from nozzle by high viscosity of the paste [21], and strong coagulation in the water, as seen from the overlapped surface layers in Figure 3(a2).

The CNTs demonstrate different thermal conductivity values from the thermal conductivity of 0.1 W/mK to high values as 6600 W/mK depending on their structure and measurement methods [24]. Examination of the thermal conductivity of the CNTs has been challenging because of various parameters considered in the tests. In this study, the thermal conductivities of the raw CNT and the prepared microbeads were measured by the light flash method to obtain the value of fixed bed or bulk state of the CNTs considering the fluidized bed application, and the obtained values were compared to see how the conductivity increased with the bead preparation in the study. Table 2 compares the measured thermal properties of the raw CNT powder and the CNT microbeads. The heat capacities (C_ps_) of the microbeads were higher than the raw CNT, and the thermal conductivity I of the microbeads was about 37 times higher than the raw powder, indicating that the thermal conductivity of CNTs is greatly affected by the nanotubes structure. As shown in the table, the raw CNT powder exhibited a low thermal conductivity value, as it was measured in the bulk or loosely packed states of the CNT powder rather than thermal conductivity of a single nanotube. In the bulk state, the CNT particles are loosely contacted between each other by van der Waals forces or physical entanglement of the nanotubes [17], thereby limiting heat transfer through the heat conduction between particles. Since the CNT microbeads have the stacked layer structure of the nanotubes inside the particles, it provides a continuous conduction pathway to neighboring layers of nanotubes, so it has high internal thermal conductivity [25]. Additionally, fine particles in the voids between the microbeads provide an additional path of heat transfer between the microbeads in the bulk state, resulting in high thermal conductivity as a whole. This internal structure and shape of the microbeads are expected to provide positive effects on the heat transfer coefficient when applied to fluidized bed [1,2].

**Figure 4:**
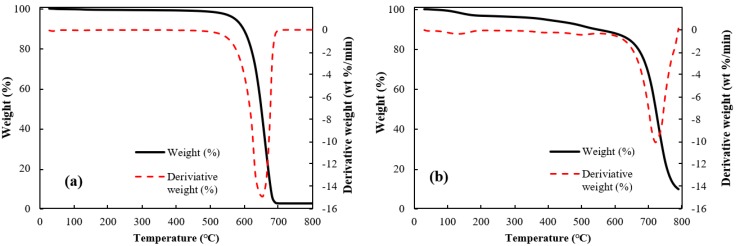
Thermal gravimetric analyses of CNTs: (**a**) raw MWCNT and (**b**) CNT microbead (case II).

Thermo-gravimetric analyses (TGA) of the raw MWCNT and the microbead (case II) having a better shape factor are shown in Figure 4. The raw CNT powder was stable at temperatures below 500 °C, but complete weight loss was observed by the CNT oxidation at 650 °C. The CNT microbead showed a gradual decrease of weight up to around 510 °C similar to the raw CNT powder, and a maximum weight loss at 700 °C. The onset of weight loss near 510 °C is due to the oxidation of non-graphitic carbon and the subsequent rapid weight loss is due to the oxidation of graphitic carbon [26]. Interestingly, the maximum oxidation temperature (peak in the derivative of weight loss) of the microbeads appeared after about 50 °C compared to the raw CNT powder. The shift of the maximum oxidation temperature of the microbeads is because the regularly stacked array structure inside the microbead obstructs the oxidation process of the CNT [26]. These results suggest that the CNT microbeads can be used stably up to about 500 °C in the air–solid fluidized bed such as a heat exchanger operated at low and medium temperature [27], considering the possibility of initial oxidation of disordered carbon.

### 3.2. Fluidization Characteristics of CNT Microbeads

**Figure 5:**
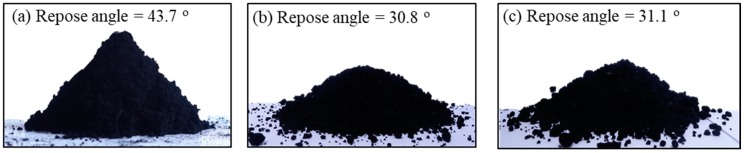
Images of the repose angle: (**a**) MWCNT, (**b**) case I and (**c**) case II.

The fluidization performance of the particles can be largely confirmed from the observation of the flowability characteristics of the particles and their behavior in the fluidized bed [1,2]. The flowability of particles depends on the repose angle of particles [1]. The angle of repose is an important intrinsic characteristic of powder rheology [2]. The poor flowability of nanopowder with a large surface area is still the biggest challenge in the application [28]. Therefore, the repose angle should be controlled by numerous factors or methods especially for the application of the fluidized beds [29]. The repose angles of the raw CNT and the microbeads are shown in Figure 5. The raw CNT shows a very high angle of 43.7° due to their strong van der Waals force. The angle is near a cohesive class in flowability classification [30], indicating its limitation in the fluidization application [28]. However, the both CNT beads show the repose angle of about 31°, which is near very or normal free-flowing group in flowability classification. The flowability of the prepared microbeads is confirmed to be as good as fluid catalytic cracking (FCC) catalysts having a repose angle of 32°, which is one of the best particles for fluidized bed application.

**Figure 6:**
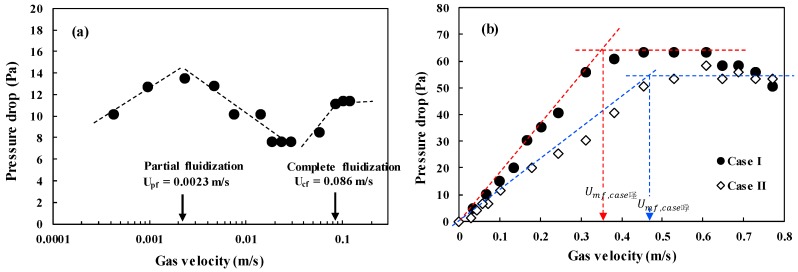
Pressure drop-versus-velocity diagram: (**a**) raw CNT powder and (**b**) CNT microbeads.

**Figure 7:**
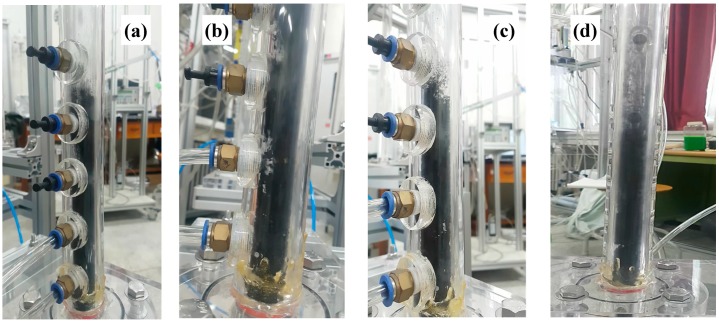
Fluidization behavior above U_mf_ of the CNT microbeads: (**a**) case I at U_mf_, (**b**) case I at U_g_ = 0.61 m/s (1.7 U_mf_), (**c**) case II at U_mf_ and(**d**) case II at U_g_ = 0.61 m/s (1.3 U_mf_**).**

It is very important to apply the prepared microbeads in the fluidized bed for confirmation of their applicability. The fluidization phenomenon of the particles can be described well by a plot of pressure drop across bed (ΔP/L) versus gas velocity [2]. Figure 6 shows the ΔP/L variation with gas velocity for determining the minimum fluidization velocity (U_mf_), which is the most important parameter associated with design and operation of the fluidized bed [31]. The pressure drops (ΔPs) across the bed increased linearly due to the friction loss and the kinetic energy loss, and reached a constant value corresponding to apparent weight of the bed materials [1,3]. However, the CNT powders experience an unusual variation of the ΔP with increasing gas velocity due to physical entanglement and strong aggregation phenomena between powders, and show unique fluidization behavior due to the van der Waals force [7,32]. The raw CNTs show a complex regime transition of partial fluidization, channeling and complete fluidization as increasing gas velocity as in Figure 6a [17,33], indicating difficulties in handling the CNT powder in a fluidized bed reactor. The CNT microbeads show variations of the pressure drop with gas velocity compared to the raw CNT. The U_mf_ values of the CNT microbeads were 0.36 m/s (case I) and 0.47 m/s (case II), respectively. The flow behavior of the prepared CNT microbeads in the study was a smooth transition from fixed bed to complete fluidization as in Figure 6b, expecting an easy operation. The flow regime of both microbeads became a bubbling fluidization immediately with further increasing gas velocity (U_g_) above the U_mf_ as observed in the bed of general Geldart’s group B particles [1] as shown in Figure 7. The vigorous bubbles in the fluidized bed enhance the mixing of the bed materials as a heat carrier, thereby make the temperature in the fluidized bed much uniform [34]. Eventually, the prepared CNT microbeads are expected to be a good candidate with good fluidity and high thermal conductivity as a bed material for the fluidized bed heat exchanger. Further research is needed to control the particle size and improve the sphericity through the application of various surfactants to make them more suitable for fluidized bed operation at lower gas velocities. In addition, it is required to improve the thermal behavior of the microbeads through further processing such as calcination.

## 4. Conclusions

A preparation method of CNT microbeads was proposed to be suitable for the fluidized bed applications. The method is characterized by using *m*-cresol known as processing solvents for the fabrication of the MWCNT microbeads. The obtained microbeads have diameters ranging 300–2200 μm and apparent particle density of 350–400 kg/m^3^, which corresponds to Geldart group B in the fluidization classification. The micrograph of the CNT microbeads exhibited obvious densification of the raw CNT powders with a better shape factor such as circularity and solidity. The thermal conductivity of the CNT microbeads was about 1.18 W/mK. The repose angle and fluidization behavior of the CNT microbeads showed a possibility as promising bed material for the fluidized bed heat exchanger.

## Figures and Tables

**Table 1:** Physical properties of raw multiwalled CNT (MWCNT) powder and prepared CNT microbeads.

Type	MWCNT Powder	Case I	Case II
Particle Density (kg/m^3^)	31	400 (0.43) ^d^	352 (0.93) ^d^
Bulk Density (kg/m^3^)	19	182 (0.54) ^d^	179 (0.47) ^d^
Particle Diameter, d_p_ (μm)	311 ^a^	1600 ^b.^	1855 ^b.^
		921 ^c.^	956 ^c.^
Circularity^.^ (-)	0.60	0.799 (3.6) ^d^	0.843 (7.6) ^d^
Solidity^.^ (-)	0.825	0.888 (1.8) ^d^	0.910 (1.7) ^d^

^a^ Measured by particle size analyzer (LA-950 V2, Horiba); ^b.^screening method; ^c^ measured by SEM images and ^d^ % relative standard deviation.

**Table 2:** Comparison of thermal properties of CNT powder and CNT microbeads.

Type	MWCNT Powder	Case I	Case II
Thermal Conductivity (Bulk Basis), k_s_ (W/mK) ^a.^	0.032	1.18 (0.87) ^c^	1.17 (1.25) ^c^
Heat Capacity, C_ps_ (J/kg K) ^b.^	721	979 (0.04) ^c^	978 (0.04) ^c^

^a^ Laser Flash Apparatus, (LFA 467, Netzsch, Germany); ^b^ Ultra Low Temp Differential Scanning Calorimetry, DSC 214 Polyma, Netzsch, Germany) and ^c^ % relative standard deviation.
